# Update on the Protective Molecular Pathways Improving Pancreatic Beta-Cell Dysfunction

**DOI:** 10.1155/2013/750540

**Published:** 2013-05-02

**Authors:** Alessandra Puddu, Roberta Sanguineti, François Mach, Franco Dallegri, Giorgio Luciano Viviani, Fabrizio Montecucco

**Affiliations:** ^1^Department of Internal Medicine, University of Genoa, Viale Benedetto XV 6, 16132 Genova, Italy; ^2^Division of Cardiology, Geneva University Hospitals, Faculty of Medicine, Foundation for Medical Researches, Avenue de la Roseraie 64, 1211 Geneva 4, Switzerland; ^3^First Medical Clinic, Laboratory of Phagocyte Physiopathology and Inflammation, Department of Internal Medicine, University of Genoa, Viale Benedetto XV 6, 16132 Genova, Italy

## Abstract

The primary function of pancreatic beta-cells is to produce and release insulin in response to increment in extracellular glucose concentrations, thus maintaining glucose homeostasis. Deficient beta-cell function can have profound metabolic consequences, leading to the development of hyperglycemia and, ultimately, diabetes mellitus. Therefore, strategies targeting the maintenance of the normal function and protecting pancreatic beta-cells from injury or death might be crucial in the treatment of diabetes. This narrative review will update evidence from the recently identified molecular regulators preserving beta-cell mass and function recovery in order to suggest potential therapeutic targets against diabetes. This review will also highlight the relevance for novel molecular pathways potentially improving beta-cell dysfunction.

## 1. Introduction

Pancreatic beta-cells are principally responsible for the transcription of the gene encoding insulin and the subsequent processing and secretion of insulin in response to increases in extracellular glucose concentrations [1]. Their dysfunction induces profound metabolic consequences, leading to the development of hyperglycemia and, ultimately, diabetes mellitus [2]. In type 2 diabetes, the reduction in beta-cell function is associated with the loss of glucose-stimulated insulin secretion (GSIS) and the reduction of beta-cell mass [3, 4]. Insulin secretion is a complex mechanism with multiple points of regulation [5]. Briefly, glucose is transported into beta-cells by the high-capacity glucose transporter (GLUT) and metabolized by glucokinase that generates glucose-6-phosphate. Furthermore, glycolytic and oxidative metabolism of glucose results in the elevation of the cytosolic ATP/ADP ratio, which drives to blockade of the ATP-sensitive potassium channels. The inhibition of the ATP-sensitive potassium channels causes the depolarization of the plasma membrane that triggers the opening of the voltage-gated calcium channels. The increased intracellular calcium concentration allows the fusion of insulin-containing granules with plasma membrane and the subsequent release of stored insulin through interactions with Ca^2+^-sensitive proteins. The loss of acute GSIS is accompanied by marked changes in beta cell phenotype and changes in gene and protein expression [6–8]. Although the cause of this metabolic deterioration is unknown, several hypotheses have been proposed. The worsening of beta-cell function over time creates a vicious cycle by which metabolic abnormalities impair insulin secretion, which further aggravates metabolic perturbations [9–11]. Indeed, the diabetic milieu is enriched with high levels of glucose, advanced glycation end-products (AGEs), proinflammatory cytokines, free fatty acids, and other lipid intermediates [12, 13]. These factors are toxic for beta-cells and might activate several stress response pathways, including oxidative and endoplasmic reticulum (ER) stress, mitochondrial dysfunction, apoptosis, and necrosis [14]. 

The molecular pathways regulating insulin secretion are also implicated in the beta-cell turnover. Therefore, the more recent research field indiabetes focused on therapeutic approaches to recover both beta-cell function and preservation as a strategy to reverse the metabolic consequences of insulin deficiency. Here, we reviewed the emerging evidence regarding molecular pathways that might be involved in improving beta-cell dysfunction. 

## 2. Soluble Extracellular Molecules and Drugs Improving Beta-Cell Function

### 2.1. Glucagon-Like Peptide-1 (GLP-1)

The decline in beta cell function in type 2 diabetes is in directly associated with impaired action of the incretin hormones, glucose-dependent insulinotropic polypeptide (GIP), and GLP-1. These hormones are secreted from the intestine in response to energy intake and glucose and may potentiate as much as 70% of the meal-induced insulin response in healthy individuals. The impairment of GLP-1 secretion is one of the most relevant pathophysiological alterations in T2DM. The recently developed treatment against diabetes, based on GLP-1 receptor agonists or dipeptidyl peptidase-4 (DPP4) inhibitors (the enzyme responsible of GLP-1 inactivation), have been shown to induce beneficial effects on beta cell function [15–21]. These biological effects of GLP-1 are selectively mediated by binding to its receptor, GLP-1R, a specific seven-transmembrane receptor guanine nucleotide-binding protein (G-protein) coupled receptor (GPCR), which is widely distributed in pancreatic islets, brain, heart, kidney, and the gastrointestinal tract [22]. In the pancreas, GLP-1 has been shown to potentiate glucose-induced insulin secretion, improve pancreatic beta-cell neogenesis and proliferation, reduce beta-cell apoptosis, inhibit glucagon secretion from pancreatic alpha-cells, restoring glucose homeostasis [23, 24]. GLP-1 also acts synergistically with glucose to promote insulin gene transcription, mRNA stability, and biosynthesis, increasing the expression of the transcription factor Pancreas duodenum homeobox-1 (Pdx-1) and the binding of this factor to the insulin promoter. Furthermore, GLP-1 confers glucose sensitivity to glucose-resistant beta-cells, thereby improving their capacity to sense and respond to glucose. The cyclic AMP (cAMP) signaling pathway is central in transducing GLP-1-mediated activities in beta-cells. In fact, GLP-1 has been shown to improve both beta-cell proliferation and survival via the cAMP-dependent stimulation of the cAMP response element-binding protein (CREB) [25–27]. These promising results were confirmed *in vivo* in obese diabetic (db/db) mice. The prolonged treatment with GLP-1 enhanced not only insulin secretion, but also beta-cell neogenesis and islet size [28]. Accordingly, Buteau and coworkers demonstrated that beta-cell apoptosis induced by gluco- and lipotoxicity was prevented by GLP-1 treatment [29]. Recently, evidence from our laboratory confirmed that GLP-1 counteracted the detrimental effects of advanced glycation end-products (AGEs) on pancreatic beta-cells, preserving both function and survival [30]. In particular, we showed that GLP-1 ameliorated glucose-induced insulin secretion and antioxidant defense and restored expression of transcriptional factors that regulate insulin gene expression.

Considering that GLP-1 is rapidly inactivated by the ubiquitous proteolytic enzyme DPP4, its therapeutic use has been partially limited. More recently, the concomitant administration of DPP4 inhibitors or the identification of more stable exogenous GLP-1R agonists has improved clinical efficacy of incretin treatments [31–35]. In particular, DPP4 inhibition has been shown to approximately double the circulating GLP-1 levels, while synthetic agonists mimicking GLP-1 action resulted in striking elevations of GLP-1 signaling [36].

### 2.2. Metformin

Metformin is an antidiabetic drug commonly used since the 1960s to treat type 2 diabetes [37]. The oral absorption, hepatic uptake, and renal excretion of metformin are mediated by organic cation transporters (OCTs) [38, 39]. The glucose-lowering properties are primarily due to the reduction in the hepatic glucose production and increase in insulin-stimulated glucose uptake within the muscle and fat tissues. These effects are mediated by the activation of AMP-activated protein kinase (AMPK) [40] and the inhibition of complex 1 in the mitochondrial respiratory chain [41, 42]. Metformin is widely used in combination with both DPP4 inhibitors and GLP-1 agonists to further suppress the hepatic production of glucose. Maida and coworkers demonstrated that metformin enhanced *in vitro* the expression of GLP-1R, via a peroxisome proliferator-activated receptor- (PPAR-) *α*-dependent pathway, improving incretin-mediated bioactivity [43]. Moreover, in rat pancreatic islets whose secretory function has been impaired by the chronic exposure to elevated FFA or glucose levels, metformin was able to restore a normal insulin secretory pattern [44]. On the other hand, the incubation of isolated T2D islets with metformin was associated with increased insulin content and release and reduced apoptosis [45]. Kefas and colleagues demonstrated that metformin dose-dependently also activates AMPK in insulin-producing MIN6 cells and in primary rat beta-cells, leading to increased phosphorylation of acetyl coA carboxylase (ACC) [46]. This chronic stimulation reduced the secretory and synthetic responsiveness of rat beta-cells to glucose and resulted in a progressive increase of apoptosis due to metformin-activated c-Jun-N-terminal kinase (JNK) and caspase-3 [47]. These controversial effects on insulin secretion and susceptibility to apoptosis might be related to the high drug concentrations that are not achieved *in vivo *[48, 49].

### 2.3. Thiazolidinediones

The thiazolidinediones (pioglitazone, rosiglitazone, and troglitazone), also known as glitazones, are a class of drugs used in the treatment of type 2 diabetes mellitus, which acts by activating the group of nuclear receptors peroxisome proliferator-activated receptors (PPARs), with greatest specificity for PPAR*γ* [50]. After activation, these receptors bind to DNA in complex with the retinoid X receptor (RXR), thus regulating transcription of several specific genes. The major clinical impact of thiazolidinediones is to improve insulin sensitivity, thereby increasing glucose uptake and reducing hepatic glucose output [51]. In addition to the insulin sensitizing effect, there is growing body of evidence indicating that thiazolidinediones preserve pancreatic beta-cell mass and function [52]. Activation of PPAR*γ* protects pancreatic beta-cells from cytotoxicity preventing nuclear factor-*κ*B (NF-*κ*B) activation [53–55]. On the other hand, pioglitazone has been shown to improve insulin secretory capacity in both diabetic humans and mice [56, 57]. In particular, treatment with this drug prevented the loss of beta-cell mass in murine diabetes and preserved human islets against glucose-induced apoptosis [58–60]. Rosiglitazone was shown to protect pancreatic islets from the apoptosis in the presence of islet amyloid polypeptide fibrils via a PI3K-Akt pathway [61]. Treatment with pioglitazone prevented the loss of the sarcoendoplasmic reticulum Ca^2+^ ATPase (SERCA) pump induced by high glucose concentration and proinflammatory cytokine interleukin-1*β* (IL-1*β*), thus favoring the maintenance the intracellular Ca^2+^ homeostasis in the pancreatic beta-cells [62]. Recently, we showed that treatment with pioglitazone restored the redox balance, improved the responsiveness to low glucose concentration, and protected cells against apoptosis and necrosis in pancreatic beta-cells exposed to advanced glycation end-products (AGEs) [63]. 

## 3. Intracellular Targets: Cytosolic Molecules Protecting Beta-Cell Homeostasis

### 3.1. Akt

The serine/threonine kinase Akt, also known as protein kinase B (PKB), is a major downstream effector of the phosphoinositide 3-kinase (PI3K) signalling pathway, activated by numerous growth factors and hormones such as insulin [64]. Akt activity consists of multiple steps that involve membrane translocation and phosphorylation [65]. Akt/PKB translocation to the nucleus results in phosphorylation of many substrates that control various biological processes. Activation of Akt signaling in transgenic mice constitutively overexpressing activated Akt in beta-cells resulted in increased islet mass, largely due to neogenesis and proliferation of beta-cells, and improved glucose tolerance [66, 67]. Alterations in Akt signaling play an important role in beta-cell adaptation to the increase of insulin demand. Indeed, reduction of Akt activity in transgenic animals expressing a kinase-dead mutant of Akt in beta-cells resulted in impaired glucose tolerance due to defective insulin secretion [68]. PI3K/Akt signalling might be a converging pathway in the regulation of beta-cell mass by growth factors, insulin, incretins, and glucose [69]. Maintenance of beta-cell mass results predominantly from proliferation of preexisting beta-cells and required the activation of the cyclins D2, and D1 [70–72]. Indeed, Akt activates beta-cell proliferation in a cdk4-dependent manner by inducing cyclin D1, D2 and p21Cip1 [73]. It is well known that the IRS2/PI3K/Akt signaling pathway is a crucial regulator of beta-cell mass and function [74, 75]. Therefore, the serine-threonine kinase Akt might represent one of the potential targets to improve beta-cell proliferation and survival. Protective incretins may activate Akt by induction of PI3K signalling in INS-1 cells and islets [76, 77]. The mechanisms involved in this process have been partially elucidated, but there is evidence that GLP-1 might promote transactivation of the epidermal growth factor receptor (EGFR) [78, 79]. GLP-1 receptor agonists could also indirectly modulate Akt signalling by activating cAMP-dependent pathways leading to the final increased transcription of IRS2 [80]. Several studies using beta-cell lines have shown the importance of Akt also for cell survival. *In vitro* experiments using insulinoma cell lines and isolated islets demonstrated that Akt activation by glucose, insulin, insulin growth factor (IGF)-1, and GLP-1 is a major determinant for the antiapoptotic effects of these molecules [23, 81, 82]. The expression of a constitutively active form of Akt in INS1 cells prevented free fatty acid-induced apoptosis and modulates survival to ER stress [83]. 

### 3.2. Molecules Involved in Unfolded Protein Response

As the main function of beta-cells is the production and secretion of insulin, the endoplasmic reticulum (ER) is very well developed and highly active in order to produce insulin even under high demand. However, these properties also likely increase the susceptibility of these cells to ER stressors, which might produce signals mediating glucose-induced impairment of function and death. Increasing experimental evidence suggests ER stress to be a relevant cause in the progressive beta-cell failure and apoptosis [84–86]. When malfolded proteins accumulate within their ER, eukaryotic cells trigger an unfolded protein response (UPR) or ER stress response, leading to an increase of chaperone gene transcription [87, 88]. The main purpose of the UPR is to restore ER homeostasis by decreasing protein load and increasing its folding capacity. The signaling pathways engaged following ER stress are complex and involve three transmembrane stress sensors: activating transcription factor 6 (ATF6), inositol requiring-1 (IRE1), and double-stranded RNA-activated protein kinase- (PKR-) like kinase (PERK), that activate genes increasing the protein folding capacity and concomitantly decrease the load of proteins entering the ER [89–91]. Indeed, the modulation of the expression of ER chaperones and the use of exogenous chemical chaperones may represent useful strategies in counteracting the ER stress pattern [92–96]. The identification of pathways alleviating beta-cell ER stress might have a considerable clinical impact on diabetes. Interestingly, it has been found that GLP-1R agonists potentiate expression of gene products subjected to the UPR in response to ER stress. Indeed, the activation of GLP-1R has been shown to stimulate the PERK arm of the UPR in both rat primary beta-cells and INS-1 cells, thus favoring beta-cell adaptation to metabolic and cellular stress [97]. In particular, the GLP-1R agonist exendin-4 attenuated the translational downregulation of insulin and improved *in vitro* cell survival following ER stress, thus shifting from translational repression to the recovery phase [98]. Furthermore Cunha and coworkers demonstrated that Exendin-4 protected pancreatic beta-cells from ER stress increasing the expression of binding immunoglobulin-protein (Bip), a key ATF6-dependent ER chaperone [97].

### 3.3. Calcium

The divalent cation Ca^2+^ plays an important role in several aspects of the beta-cell physiology. GSIS occurs through a Ca^2+^-dependent mechanism coupling cellular depolarization with cytosolic Ca^2+^ influx from voltage-gated Ca^2+^ channels and insulin granule exocytosis [99, 100]. In addition to this central role in the secretory response, Ca^2+^ homeostasis is important in ER function, including protein folding and maturation [101, 102]. In particular, the maintenance of a robust pool of Ca^2+^ in the ER plays a key role in several aspects of beta-cell function including insulin production and secretion and the maintenance of ER health. The flux of Ca^2+^ across the ER is regulated by SERCA2b, a pump resident in the ER membrane, that hydrolyzes one ATP molecule to move two Ca^2+^ molecules across the sarco- or ER membrane [103]. Inhibition of SERCA2b reduced the efflux of Ca^2+^ from the ER and leads to activation of the UPR [104]. Evidence that SERCA2b expression in pancreatic beta-cells is decreased in selected models of diabetes has emerged from several studies [105–107]. Moreover, SERCA2b is downregulated in rodent diabetic or human islets isolated from cadaveric T2DM diabetic donors [62]. *In vitro* experiments demonstrate that the thiazolidinedione pioglitazone preserves SERCA2b expression in the presence of high glucose concentrations (25 mM) and inflammatory cytokines, through modulation of cyclin-dependent kinase 5 activity and PPAR-*γ* phosphorylation [62]. Expression of SERCA is also preserved by exendin-4 through a PKA-dependent pathway [108]. 

### 3.4. Glucokinase

Glucokinase (GK) is an enzyme that phosphorylates glucose [109]. Since GK activity has been related to the induction of insulin secretion, GK is considered as a “glucose sensor” in pancreatic beta-cells. It has been reported that a mutation in GK can lead to maturity onset of diabetes mellitus in young (MODY) [110, 111]. Moreover, GK^+/−^ mice showed decreased beta-cell replication and impaired insulin secretion in response to glucose, suggesting that GK regulates pancreatic beta-cell mass as well as their function [112]. The induction of the glycation reaction, which is known to occur in pancreatic beta-cells in chronic hyperglycaemia, suppresses the glucokinase gene transcription and its enzymatic activity [113]. Therefore, pancreatic beta-cell function may be improved by the activation of GK. The identification of GK pharmacological activators has started in 2001 and showed from the beginning a high potential to improve current treatment of type 2 diabetes mellitus [114]. In addition, posttranslation activation of GK is an important mechanism for mediating the insulinotropic effects of GLP-1 [115]. Moreover, exedin-4 has been shown to stimulate GK expression within the pancreatic beta-cell line INS-1 via a Ca^2+^/calmodulin- (CaM-) dependent protein kinase cascade [116].

### 3.5. Reactive Oxygen Species (ROS)

The dynamic, fluctuating activation of stress signalling is required for the maintenance of survival, whereas its persistent activation results in dysfunction and apoptosis of pancreatic beta-cells. The relatively low expression and activity of many enzymes involved in the antioxidant defense renders beta-cells highly susceptible to oxidative damage [117]. In particular, reactive oxygen species (ROS, such as superoxide anion and hydrogen peroxide), might contribute to beta-cell dysfunction [118–120]. ROS may originate during cellular metabolic processes or may be introduced via toxic extracellular mediators. Mitochondria are an important intracellular source of ROS, and, in turn, also a target of ROS-mediated injury. Superoxide anion is a very reactive molecule, which can be converted to less reactive H_2_O_2_ by superoxide dismutase (SOD) isoenzymes, and then to oxygen and water mainly by catalase (CAT), glutathione peroxidases (GPxs), and peroxiredoxin [121]. The levels of the H_2_O_2_-inactivating enzymes GPxs and CAT are extremely low in pancreatic beta-cells [117]. Therefore, their defense against ROS toxicity is very limited. The transcription factor NF-E2-related factor 2 (Nrf2) [122] is one of the major antioxidant pathway allowing the synthesis of many protective enzymes [123]. Accumulating evidence suggests important connections between Nrf2, PPAR*γ*, and PI3K/Akt on regulating antioxidant enzymes in diabetes [124]. Glutathione remains the most important intracellular defense against ROS [125, 126], implying that the ratio of the oxidized form of glutathione (GSSG) and the reduced form (GSH) is considered as a dynamic indicator of the oxidative stress of an organism. It has also been reported that glucose metabolism might increase the ROS-scavenger potential of the pancreatic beta-cells through generation of NAD(P)H, and that this effect seems to be more pronounced in beta-cells with higher metabolic responsiveness to glucose [127].

In pancreatic beta-cells, ROS generation is not only a harmful process but, rather, plays a substantial role in the normal insulin signal transduction and might be one of the metabolic signals stimulating insulin secretion. Indeed, in pancreatic beta-cells ROS generation might also occur in response to glucose stimulation [128, 129], as a consequence of glycolytic and oxidative events leading to accelerated ATP generation. In particular, Pi and coworkers demonstrated that glucose-induced intracellular H_2_O_2_ accumulation coincides with glucose-stimulated insulin secretion [130]. Considering that the role of H_2_O_2_ in glucose-stimulated insulin secretion is controversial, Lortz and colleagues recently reported that the overexpression of the H_2_O_2_ inactivating enzyme catalase did not affect insulin secretion in response to glucose [131]. These discrepancies might be explained by the different time of incubation and culture conditions. Indeed, the study by Leloup and coworkers showed that transient mitochondrial ROS production is required for glucose-induced insulin secretion [132]. This implies that the ROS levels need to be finely regulated to keep “good” instead of “bad” radicals, thus avoiding oxidative damages. 

### 3.6. MicroRNAs

MicroRNAs (miRNAs) are small nucleotide noncoding RNA molecules, which regulate gene expression by inhibiting translation or inducing target mRNA degradation [133–135]. The role of miRNAs in beta-cell mass regulation is not fully understood. However, it has been suggested that miRNAs target genes are important for pancreas development, beta-cell proliferation, insulin secretion, and exocytosis [136]. Several recent studies suggested that the modulation of miRNA expression could be of interest for novel treatments against diabetes. MicroRNA-375 (miR-375) is necessary for the proper formation of pancreatic islets in vertebrates and is necessary for the development of beta-cells in mice [137, 138]. The specific knockdown of miR-24, miR-26, miR-182, or miR-148 in cultured beta-cells or in isolated primary islets downregulated insulin promoter activity and mRNA levels [139, 140]. Among different miRNAs, mir-9 has been shown to regulate exocytosis in beta-cells [141]; miR-30d induced insulin gene expression in pancreatic beta-cells, associated with increased expression of MafA, a beta-cell specific transcription factor [142]. On the other hand, the overexpression of miR-21 downregulated proteins involved in insulin secretion [143], while the overexpression of other miRNAs (such as miR-33a, miR-375, and miR-29a/b/c), lead to impairment in glucose-induced insulin secretion [144–146]. 

## 4. Nuclear Factors

The specialized features of beta-cells are determined by the expression of a gene subset controlled by a variety of transcription factors. Insulin production is achieved by a strict regulation of insulin synthesis and exocytosis at the transcriptional and posttranscriptional levels, mainly regulated by blood glucose concentration [147–149]. The activity of beta-cell transcription factors is modulated at a multiple upstream level including subcellular localization, DNA-binding activity, transactivation capability, and interaction with other proteins. 

Among the numerous transcription factors implicated in the regulation of insulin transcription, V-maf musculoaponeurotic fibrosarcoma oncogene homolog A (MafA), Pancreatic and duodenal homeobox 1 (Pdx-1), forkhead box protein O1 (FoxO1), and nuclear factor E2-related factor 2 (Nrf2) have been demonstrated to play a crucial role in pancreatic beta-cell function [147, 149].

## 5. MafA

MafA is a basic leucine zipper transcription factor belonging to the large Maf family of transcription factors. In pancreatic beta-cells, MafA has been shown to play an important role in glucose regulation of insulin gene expression and in mediating the expression of a number of other genes, including PDX-1 [149–151]. MafA levels in beta-cells might be regulated by posttranscriptional mechanisms, such as the phosphorylation of two residues (serines 14 and 65) located in the transcriptional activating domain by the mitogen-activated protein kinase 1 (also known as MAPK1 or ERK2). The expression of MafA is observed at later stage of beta-cell development suggesting a role for MafA as crucial master regulator of genes implicated in maintaining beta-cell function in response to glucose [152].

MafA knockout mice are viable, but develop diabetes during life as a result of the decreased insulin secretion from beta-cells and alteration of islet architecture [147, 152, 153].

Recently, biochemical studies revealed that FoxO1 and PDX-1 bind directly to the MafA promoter and mediate MafA transcription [149], suggesting that different transcription factors might regulate insulin regulation within beta-cells.

Enhanced production of MafA under high-glucose concentrations may regulate the glucose-dependent transcription of the insulin gene, whereas decreased production or proteasomal degradation of MafA probably rapidly inhibited insulin transcription. These results suggest that MafaA upregulation within beta-cells should precede the insulin transcription process [153]. The downregulation of the expression of MafA gene has been observed in the presence of lipotoxicity as well as exposure to proinflammatory cytokines [152, 154, 155].

All these results indicate that MafA may be qualified as a crucial master regulator of genes implicated in maintaining beta-cell function and glucose-stimulated insulin synthesis. Its modulation may represent a key therapeutic target to prevent beta-cell dysfunction in response to injury [152].

## 6. PDX-1

The pancreatic and duodenal homeobox 1 (PDX-1) is critical in both beta-cell development and function [149, 152]. Similar to MafA, it is considered as a major regulator of glucose-stimulated insulin gene transcription. PDX-1 could be regulated at the transcriptional, posttranscriptional, and translational level and its expression is conditioned by several mechanisms of beta-cell damage (such as glucotoxicity, lipotoxicity, oxidative stress, and inflammation) [147]. The subcellular localization of PDX-1 has been shown to be regulated by the changes in glucose levels. When exposed to low glucose concentrations, PDX-1 is mainly localized to the nuclear periphery and associated with histone deacetylase-1 and -2 (HDAC-1 and HDAC-2) and does not interfere with insulin gene expression. In the presence of increased glucose levels, PDX-1 promotes shuttle to the nucleoplasm of its phosphorylated form and becomes associated with the histone acetyltransferase (HAT) p300, leading to hyperacetylation of histone H4 and induction of insulin gene transcription [147, 156, 157].

Therefore, the downregulation of PDX-1 importantly affects insulin production favoring beta-cell secretory dysfunction and potentially diabetes [158]. As an example, ROS have been described to potently inhibit PDX-1 in beta-cells [159–162]. Indeed, various studies have shown that oxidative stress inhibits Pdx-1 nuclear localization and DNA binding through the activation of the c-Jun N-terminal kinase (JNK) pathway [149, 163].

As reported by Robertson and coworkers using a pancreatic islet cell line HIT-T15, the generation of ROS might cause the loss of PDX-1 protein as a consequence of the posttranscriptional loss of PDX-1 mRNA [12, 164]. More recently, Tingting and colleagues reported that the exposure to AGEs in INS-1 cells decreased PDX-1 protein levels without a decrement in PDX-1 mRNA level and promoter activity. Therefore, PDX-1 protein expression deficiency might be not due to the inhibition of its transcription, but potentially to the decrease in its protein stability related to its nucleocytoplasmic translocation [158, 165].

Several studies suggest that PDX-1 may serve as a target for other posttranscriptional and posttranslational modifications, such as glycosylation. Gao and coworkers demonstrated that PDX-1 was also modified by O-linked N-acetylglucosamine (O-GlcNAc), with a consequent positive association between protein O-Glc-NAcylation, PDX-1 DNA-binding activity, and insulin secretion. These data indicate that this ubiquitous nucleocytoplasmic saccharide modification might participate to the regulation of insulin gene expression and it may be involved in the development of insulin resistance within the beta-cells or peripheral tissues [166].

As discussed above, GLP-1 agonists have been shown to improve beta-cell morphology and function. GLP-1-mediated pathways of beta-cell protection can act at nuclear level involving the regulation of PDX-1. Indeed, GLP-1 directly increased PDX-1 levels and its nuclear localization, enhancing its DNA-binding activity [26, 167, 168].

Importantly, Shao and colleagues showed that the abrogation of PDX-1 expression in INS-1 cells downregulated GLP-1R levels, triggering a vicious circle, which might contribute to beta-cell dysfunction. These data demonstrate that PDX-1 plays a key role in GLP-1/GLP-1R pathway and in glucose-stimulated insulin synthesis, thus representing a beta-cell protective target at a nuclear level [149, 167].

## 7. Foxo-1

Forkhead transcription factors FOX of the O subfamily belonging to the large family of Forkhead proteins play an important role in cellular differentiation, proliferation, metabolism, and stress resistance. 

FoxO1, the most predominantly expressed FoxO factor in beta-cells, is a prominent mediator of growth factor signaling, and is required to regulate both replication and response to oxidative stress in beta-cells [169, 170]. 

FoxO1 is normally present as a cytoplasmic phosphorylated form in healthy beta-cells; when mild hyperglycemia occurs, this factor is localized in the periphery of the nucleus. At severe hyperglycemic levels and/or oxidative stress, FoxO1 undergoes nucleoplasm translocation. Within the nucleus, it activates a transcriptional program to preserve insulin secretion, decreasing intracellular glucose metabolism with the activation of free fatty acid oxidation [165, 171, 172]. 

Under different conditions, FoxO1 has been shown to induce either protective or proapoptotic functions in beta-cells. FoxO1 may contribute to cellular responses against oxidative stress inducing antioxidant enzymes catalase and superoxide-dismutase and play important roles to prevent somatic mutations induced by DNA damage. 

On the other hand, beta-cell exposure to oxidants results in the nuclear redistribution of FoxO1, associated with increased expression of a well-known insulin gene transcription factor, such as MafA [173]. 

In the pathogenesis of type 2 diabetes, FoxO1 has been shown to control beta-cell compensation of insulin resistance through cell proliferation and mass regulation. This process requires FoxO1 nuclear exclusion and is associated with increased expression of PDX-1 [169, 174]. 

On the other hand, FoxO1 might regulate also beta-cell mass through the integration of the proliferative and antiapoptotic signals of beta-cell growth factors. This property occurs as a result of FoxO1 inhibition through its phosphorylation-dependent nuclear exclusion. The removal of FoxO1 from the nucleus allows the expression of the main beta-cell transcription factor (such as PDX-1), which is negatively regulated by FoxO1 [175, 176].

Under chronic high glucose concentration exposure, FoxO1 has been shown to shuttle to the nucleus in a dephosphorylated form. The translocation and interaction between FoxO1 and PDX-1 represents a pivotal strategy of cellular defense to preserve the insulin synthesis that would otherwise be excessive leading to cell exhaustion.

FoxO1 might be a general regulator of beta-cell mass also in response to incretins and their analogues. Several studies reported that the GLP-1-dependent proliferation and antiapoptotic actions in beta-cell depend on FoxO1 inhibition due to its phosphorylation-dependent nuclear exclusion as a consequence of PI 3-kinase/Akt signaling cascade activation [79, 177].

In type 2 diabetes, lipotoxicity plays an essential role in inducing pancreatic beta-cell apoptotic pathways that initiate mitochondrial perturbation and increase oxidative stress [178]. Recently, it has been shown that, *in vitro* in palmitate-treated INS-1 cells, the phosphorilation of Akt and FoxO1 is decreased and the pretreatment with geniposide, a new agonist for GLP-1R, reversed this phenomenon increasing PDX-1 levels [61, 179].

FoxO1 is also an important specific transcription factor required for the maintenance of cellular identity. Recently, Talchai and coworkers demonstrated that under chronic pathophysiologic stress, beta-cells undergo dedifferentiation in nonpancreatic endocrine cells with an acquired loss of FoxO1. During metabolic stress, FoxO1 is required to limit beta-cell fate by promoting genes required for beta-cell identity and by preventing reactivation of embryonic endocrine progenitor genes. The dedifferentiation phenomenon occurs commonly in type 2 diabetes and is an ordinary mechanism of beta-cell failure in different models of metabolic stress. Beta-cell dedifferentiation is a regression to an endocrine progenitor-like stage that express markers normally observed in multipotent endocrine progenitors found within the developing pancreas [180]. Indeed, dedifferentiation is associated with an impressive upregulation of specific markers of pluripotency and cellular reprogramming, such as Neurog 3, Oct 4, Nanog, and L-Myc. These cells appeared as “degranulated” with a decreased insulin content associated to an acquired loss of FoxO1 function. However, the expression of beta-cell markers (such as PDX-1 and MafA) was preserved [172]. 

FoxO1 ablation seems to have little effects when insulin demand is modest. In beta-cells exposed to sustained stress, the loss of FoxO1 resulted in a profound reduction of insulin producing cells due to the deconstruction of the mature beta-cell state [172].

Although these observations provide hope for the development of a treatment for beta-cell dysfunction in diabetes based on the “redifferentiation” of the beta-cells, whether such transient states might occur during diabetogenesis, and whether beta-cell regeneration would be possible, remains to be explored.

## 8. Nrf2

The oxidative stress can directly or indirectly disturb physiological functions of many cellular macromolecules such as DNA, protein, and lipids and activate cellular stress-sensitive signaling pathways [130, 181]. The induction of antioxidant/detoxification enzymes (e.g., *N*-acetylcysteine and aminoguanidine, which enhance cellular ROS-scavenging capacity) represents a key element in the maintenance of cellular redox homeostasis. Among these, the Nuclear factor E2-related factor 2 (Nrf2) is considered as a master regulator of the cellular adaptive response to oxidative stress and represents a critically important cellular defense mechanism that limits oxidative damage [181].

In response to oxidative stress, Nrf2 heterodimerizes with small Maf proteins and other basic leucine zipper proteins, binding to antioxidant response elements (AREs) in the promoters of many phase II detoxification (e.g., glutathione-*S*-transferases and NAD[P]H quinone oxidoreductase) and antioxidant genes (e.g., heme oxygenase-1, glutathione peroxidase, Cu/Zn-superoxide dismutase (SOD), and Mn-SOD) [182, 183].

In the early stages of oxidative stress, the adaptive response, primarily regulated by Nrf2, is the main mechanism upregulating antioxidants and phase II detoxification enzymes.

In the absence of an appropriate compensatory response from the endogenous antioxidant network, the oxidative stress may cause oxidative damage and activate the cell death machinery. In this regard, the abolishment of the Nrf2-mediated antioxidant response by targeted disruption of the Nrf2 gene in beta-cells due to various stress conditions (e.g., glucose starvation, oxidative stress, hypoxia, high fat or cholesterol, aberrant levels of free fatty acid, and inflammatory cytokines) perturbed the protein homeostasis, leading to the accumulation of misfolded proteins in the ER lumen [183].

Nrf2-mediated antioxidant response has been shown to play a paradoxical role in insulin secretion. Under low environmentally levels of detrimental stimuli, beta-cells can adapt to the condition adequately by activating the Nrf2-ARE system, thus minimizing oxidative damage-related impairment of insulin secretion. Under chronic exposure conditions, the adaptively increased endogenous antioxidant capacity might interfere with glucose-dependent endogenous ROS signaling leading to an excessive and detrimental decreased in glucose-stimulated insulin secretion [181, 183].

Recently, Lee and coworkers reported that Nrf2 upregulated the proteasome catalytic subunit Psmb5, leading to a novel concept that the proteasome may participate in the cellular defense against different sources of stress [184].

Nrf2, controlling a battery of protective genes, represents an important mediator, by which mammalian cells can sense and adapt to chemical and oxidative stresses. Strategies to pharmacologically manipulate the levels and/or activity of Nrf2 may have the potential to reduce pancreatic beta-cell dysfunction and increase sensitivity to antidiabetic treatments.

## 9. Potential Future Targets

Several recent studies investigated the role of miRNAs in pancreatic beta-cells, suggesting the modulation of miRNA expression to be targeted in new therapies to improve diabetes. Furthermore, considering that environmental factors and nutrition might have a pivotal role in the pathogenesis of diabetes [185], epigenetic changes in response to environmental stimuli may become a pivotal research field for future investigations [185]. Current therapeutic strategies may be further potentiated by approaches targeting these “new” factors to ameliorate pancreatic beta-cell function.

## 10. Conclusion

**Figure 1:**
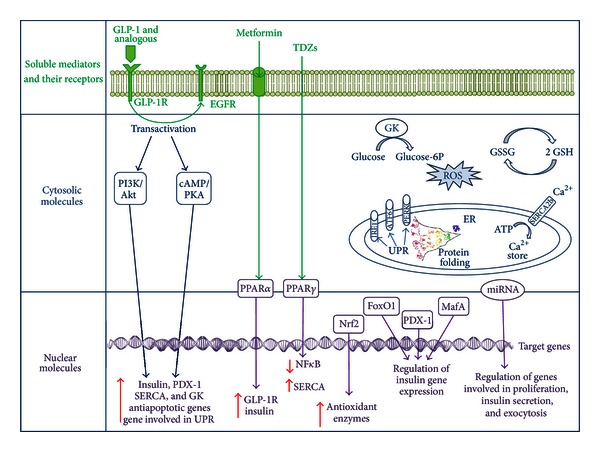
Intracellular effects of soluble mediators are mainly mediated through the interaction with specific receptors that link extracellular signals up to modulation of gene expression. *GLP-1* signaling pathway is activated after the binding to its receptor, GLP-1R, and subsequent transactivation of the EGFR. GLP-1's effects on beta-cell are mainly mediated by the cAMP signaling pathway. GLP-1 potentiates glucose-induced insulin secretion, improves the function of pancreatic beta-cells by promoting neogenesis and proliferation and by decreasing apoptosis signals, increases antioxidant defense, promotes insulin gene transcription, mRNA stability, and biosynthesis, and increases the expression of Pdx-1 and the binding of Pdx-1 to the insulin promoter. Exendin 4, a GLP-1R agonist, potentiates expression of gene products subjected to the UPR in response to ER stress, stimulates GK expression, and prevents depletion of SERCA expression. *Metformin* enhances the expression of GLP-1R, via a peroxisome proliferator-activated receptor- (PPAR-) *α*-dependent mechanism, and improves the responsiveness to incretins. The *thiazolidinediones* (TDZs) act by activating the nuclear receptors PPAR*γ*. TDZs preserve pancreatic beta-cell mass and improve their function, prevent NF-*κ*B activation, improve insulin secretory capacity in patients with diabetes, protect human islets against apoptosis, and maintain the homeostasis of intracellular Ca^2+^ preventing the loss of SERCA. Extracellular signaling is mediated by cytosolic molecules which can be enzymes, ions, and so on. The *PI3K/Akt* signaling is a converging pathway in the regulation of beta-cell mass by growth factors, insulin, incretins, and glucose. The health of the beta-cell is also related to the maintenance of its homeostasis during its intensive function. The *UPR* restores ER homeostasis by decreasing ER protein load and increasing ER folding capacity. Intracellular Ca^2+^ concentration is important for GSIS, and also for ER function. The enzyme *GK*, which is considered a “glucose sensor” in pancreatic beta-cells, regulates pancreatic beta cell mass as well as their function. The temporally fluctuating activation of stress signaling is required for the maintenance of beta-cell survival, whereas its persistent activation results in beta-cell dysfunction and apoptosis. Indeed, ROS generation is not only a harmful process but, rather, plays a substantial role in the normal insulin signal transduction and is one of the metabolic signals stimulating insulin secretion. The ROS levels need to be finely regulated to keep good radicals from going bad thus avoiding oxidative damages. The ratio of the oxidized form of glutathione (GSSG) and the reduced form (GSH) is a dynamic indicator of the oxidative stress. The cellular adaptive response to oxidative stress is finely regulated by *Nrf2*, which upregulates transcription of antioxidant and phase II detoxification enzymes. Regulation of gene transcription is regulated at different biological levels. Recently, miRNAs target genes emerged important for pancreas development, beta-cell proliferation, insulin secretion, and exocytosis. Interestingly, as expression of the transcription factors implicated in the regulation of insulin transcription, such as *MafA*, *Pdx*-1, and *FoxO*1, are controlled by the relationship between themselves for instance, FoxO1 and PDX-1 bind directly to the MafA promoter and mediate MafA transcription, and transcription of the gene coding for PDX-1 is negatively regulated by the binding of FoxO1 to the PDX-1 promoter.

The progression from glucose intolerance to type 2 diabetes is finely related with insulin secretory dysfunction and significant loss of functional beta-cells. A better understanding of the protective molecular pathways improving pancreatic beta-cell dysfunction is of primary importance to block the natural history of type 2 diabetes (Figure 1). This narrative review leads to new therapeutic targets potentially capable of preserving beta-cell function.
